# Demand- and supply-side factors associated with the use of contraceptive methods in Pakistan: a comparative study of demographic and health surveys, 1990–2018

**DOI:** 10.1186/s12905-020-01112-4

**Published:** 2020-11-30

**Authors:** Sadia Jabeen, Adnan Rathor, Maria Riaz, Rubeena Zakar, Florian Fischer

**Affiliations:** 1 Virtual University Lahore, Lahore, Pakistan; 2 Institute of Social and Cultural Studies, University of the Punjab, Lahore, Pakistan; 3 Department of Public Health, University of the Punjab, Lahore, Pakistan; 4 Institute of Public Health, Charité – Universitätsmedizin Berlin, Berlin, Germany; 5 Institute of Gerontological Health Services and Nursing Research, Ravensburg-Weingarten University of Applied Sciences, Weingarten, Germany

**Keywords:** Contraception, Demand, Supply, Pakistan, Sexual and reproductive health

## Abstract

**Background:**

A remarkable decline in fertility rates has been observed in many countries, with a primary determinant being an increase in the use of contraceptives. However, the birth rate in Pakistan is still higher compared to the other countries of the region. Therefore, this study aims to assess the effect of demand- and supply-side factors associated with the use of contraceptive measures in Pakistan.

**Methods:**

Secondary data analysis of four data series of the Pakistan Demographic and Health Surveys (PDHS 1990–1991, 2006–2007, 2012–2013 and 2017–2018) were used. The data includes ever-married women aged 15–49 years who had given birth in the previous five years and participated in the family planning module of the PDHS. A total of 25,318 women were included in the analysis. Data were analysed by investigating the associations between independent variables (demand- and supply-side factors) and the use of contraceptive measures through unadjusted odds ratios (OR) and adjusted OR (AOR).

**Results:**

The results among demand-side factors indicated that in 2012–2013, women without media exposure were less likely to use contraceptives and the trend remains almost constant for 2017–2018 (AOR = 0.664, 95% CI 0.562–0.784) in 2012–2013 and (AOR = 0.654, 95% CI 0.483–0.885) in 2017–2018. However, they still show a lower likelihood of using contraceptives without media exposure. The results among supply-side factors indicated that absence of transport (2012–2013) and limited visits by family planning workers over the previous 12 months (2006–2007, 2012–2013 and 2017–2018) remained significant factors for not using contraceptive methods.

**Conclusions:**

The results of the study indicate that certain demand- and supply-side factors are associated with the use of contraceptive measures in Pakistan. It highlights the need for the provision of family planning resources and further structural factors, particularly in remote areas.

## Background

A remarkable decline in global fertility rates has been observed over the last twenty years. The increase in the use of contraceptives in many countries can be seen as a primary determinant of declining fertility rates [1–3]. However, the decline in fertility rates is unevenly distributed, as it is less pronounced in developing countries [4, 5].

Pakistan is the sixth most populated country in the world and has one of the highest fertility rates (3.5 children per women) in the South Asian region [6]. The contraceptive prevalence rate increased from 4 to 28% between 1991 and 2000, but the rate of increase has diminished thereafter, with prevalence only rising from 30% in 2006 to 35% in 2012–2013 [7]. High fertility is an effect of the low use of contraceptives, which also hampers socio-economic development [8–10]. Although family planning programmes were launched in Pakistan during different eras, effective implementation remains challenging due to multiple socio-cultural, economic and political factors, together with a preference for male children, supply issues and the strong patriarchal structure [11].

The low use of contraceptives and associated hindrances has been explained by different studies examining various dimensions. Until now, few studies identified socio-cultural reasons (e.g. need for approval from husband and family) related to the use of contraceptives. Further studies explained the decline with geographical and financial inhibitions, going along with legal restrictions, and limitations in the availability of and access to family planning services, and associated adverse side effects that negatively impact on mother’s health [12–14]. Furthermore, misconceptions and myths associated with contraceptive use have been observed as contextual factors. The status of women, their independence in decision-making, financial autonomy, religious inspirations and cultural beliefs that high fertility is good are the factors that affect the use of contraceptives in the developing world [15–17].

Knowledge, awareness and attitudes drive the demand for contraceptive methods. When there is a demand, then there is also a compulsion on governments to provide a supply. Supply-side factors include the provision of family planning resources, health facilities and the availability of transport as an indicator of the infrastructure. A number of interventions are required to balance between the demand and supply side in the use of contraceptive measures. The failure to provide a supply to women may affect various female health issues, including unintended pregnancies and abortions [18, 19].

The influence of husbands and mothers-in-law in family matters is so strong in Pakistan that they are considered a central decision-making authority in family planning decisions as well [20]. The literature suggests that women’s own knowledge, their perception of their husband’s reaction to the use of contraceptives and their acceptability are the basic determinants of contraceptive use [21, 22]. Another important indicator in this regard is violence. A meta-analysis revealed that women who face intimate partner violence are more likely not to use contraceptive methods [23]. Similar findings have been described by Teitelman et al. [24]: Young women who do not experience intimate partner violence are more consistent in their use of condoms than abused women. Supporting this, Fanslow et al. [25] explained that the use of contraceptives is low among women who experienced violence from their partners because it claiming for the use of contraceptives might further increase the risk of emotional and verbal abuse.

The majority of family planning services are supplied in urban localities and the provision and supply of such services is insufficient in rural areas. Access to information and low-cost supply-side services facilitate women to continue their use of contraceptives. Supply-side services include the clinic location, well-equipped and trained staff, convenient opening hours, low fees and easy availability of condoms and pills [26]. A client’s satisfaction and long-term acceptance of the use of contraceptives has an impact on their decision about whether to go on with the services. Many studies have argued that the failure of family planning programmes in Pakistan is mainly due to the inadequate and limited supply of services [27].

Although previous research has identified several factors associated with contraceptive use in Pakistan, a holistic view and the inclusion of a trend analysis is still missing. Therefore, the present study uses data from four Demographic and Health Surveys in Pakistan (PDHS) in order to gain a clear understanding of the determinants for the use of contraceptives, allowing us to explore how demand and supply-side factors and socio-demographic characteristics have influenced the use of contraceptives over the years in Pakistan.

## Methods

We used a dataset series of four nationally representative PDHS (1990–1991, 2006–2007, 2012–2013 and 2017–2018). These surveys are conducted in order to gain information on mother and child health, fertility, family planning, reproductive health, and nutritional and immunisation status. So far, four standard PDHS have been conducted and data from all these surveys has been included in this research. The response rates of each PDHS of ever-married women were 6611 (95%) in 1990–1991, 10,023 (95%) in 2006–2007, 13,558 (93%) in 2012–2013 and 15,509 (96%) in 2017–2018. The surveys used a multistage sampling procedure: at the first stage, strata were built on an urban and rural basis; from each strata, households were selected by using a simple random sampling method. Our study focused on the personal, socio-cultural, community-level and supply-side factors dealing with the use of contraceptives. Women who had given birth in the previous five years and participated in the family planning module were selected to obtain the sample for this study. The selection of women as respondents was made on the basis that almost all of the family planning programs in Pakistan have remained women-focused and they are considered the main clients for any family planning interventions. The sample from each data set is as follows: 4092 women in 1990–1991, 5742 women in 2006–2007, 7461 women in 2012–2013 and 8219 in 2017–2018 [28–31].

### Instrumentation and data classification

The current use of contraceptive methods was defined as the dependent variable, including traditional (periodic abstinence [rhythm], withdrawal and abstinence) and modern methods (pill, intrauterine devices, injections, diaphragm, condom, female or male sterilisation, implants, female condom, foam/jelly and lactational amenorrhea). The reason behind combining both types of methods was that the focus of the study was to see the use and non-use of contraceptive measures rather than the types of methods being used.

Women’s socio-demographic, and demand- and supply-side factors were considered as independent variables. Women’s socio-demographic factors were: age, type of residence (urban vs. rural), region, ethnicity, education (no education, primary, secondary, higher), women’s occupation/employment status (not working, unskilled employment [sales, household domestic, unskilled manual], skilled employment [self-employed, agricultural employees, skilled manual, clerical] and professional [professional/technical/managerial services]). The wealth index of women was calculated through quintiles. Afterwards, the quintiles were categorised into three main categories (poor, middle, rich) to make it more clear and vivid for this analysis.

Among the demand-side factors, questions regarding media exposure, desire for children, number of sons living, number of daughters living, history of intimate partner violence, decisional autonomy, permission to attend medical or health facilities from male members of the family specifically husbands, as well as unwillingness to go alone and concerns about going to female health providers were included. Exposure to any source of information (TV, radio, newspaper) was computed and recoded as overall media exposure including print and electronic media, and response categories generated were “No” and “Yes”. Desire for more children was categorised into two categories: either “No” (wanted no more children, sterilised [respondent or partner]) or “Yes” (wanted within or after the next two years, unsure about timing, undecided). Intimate partner violence was part of PDHS 2012–2013, in which emotional, physical and sexual violence were included. Women were asked if they had ever faced any humiliating attitude from their husband, physical violence (such as being beaten, having their arms twisted, hair pulled) or threatened with a harmful weapon (such as a knife or gun), or sexual violence from their husbands, which includes forced sex. All forms of violence were coded as binary categories and, thereafter, combined as overall violence by intimate partners as “Yes” if faced with any type of violence and “No” if not faced with violence. However, data for this variable was not available for the previous PDHS 1990–1991 and 2006–2007. Women’s independent or joint control of income, purchases, healthcare decisions and visits to relatives were included women’s decisional autonomy. Each variable was first coded into two categories: “Yes” if the respondent contributed to any type of decision either individually or jointly, and “No” if she did not participate in any decision-making. All types were then combined into overall autonomy as women who have any type of autonomy as “Yes” and those who do not have any autonomy as “No”. The responses to questions regarding permission to attend medical or health facilities were divided into two categories (“Big problem” and “Not a big problem”). Similarly, going alone to get medical treatment was divided into the same two categories. The number of sons living has also been taken as a variable to determine whether use of contraceptives is contingent upon son preference. It has been coded as “No living son”, “1–3”, “4–6” and “7–10”.

Among supply-side factors, the facilitation and provision of governmental and non-governmental family planning services was measured through questions about the distance to health facilities, transport availability, visits by lady health workers (LHWs), unmet needs and the availability of contraceptives through different sources. The distance to health facilities and transport availability were categorised as either “Big problem” or “Not a big problem”. The sources of family planning were first categorised into public (government hospitals, family planning clinics and LHWs), private (private hospitals, pharmacies and clinics) and others (such as shops, friends or relatives and traditional practitioners). Unmet needs included those for the spacing and limiting of births. Per definition, unmet needs relate to women who do not use any contraceptive methods, although they wish to stop or limit childbearing. From 2012–2013 onwards, a revised definition has been used that includes unwanted pregnancy (in the next two years), being not sure and having postpartum amenorrhea for up to two years following an unwanted birth.

### Statistical analysis

Data was analysed using SPSS version 24. Absolute numbers and weighted percentages were obtained through descriptive analysis. The purpose of weighting was to balance the data to reflect the population more accurately that can project the result of the large universe of this study. The relationship between demographic characteristics, and demand- and supply-side factors, along with current use and non-use of contraceptives was assessed through the Chi square test (X^2^) on categorical variables. A *p* value of < 0.05 was considered statistically significant. Associations between demand- and supply-side indicators of non-use of contraceptives were measured using binary logistic regression models to present odds ratios (OR). Multivariable analysis was conducted by assessing adjusted ORs (AORs) through controlling the demographic variables (age, education, income, wealth, residence). Data on ethnicity is missing in PDHS 1990–1991 and 2017–2018, so it was not included in the analysis. Furthermore, several variables relating to demand-side factors (media exposure, intimate partner violence, decisional autonomy, permission to attend medical or health facilities, not wanting to go alone for medical help) and supply-side factors (distance to the health facility, transport availability, visit by a family planning worker during the previous 12 months) were not included in the questionnaire in 1990–1991 or 2006–2007 and, therefore, were not included in the analysis for these years.

## Results

The total sample across all four surveys includes 25,415 ever-married women aged 15–49 years having children under 5 years of age. The mean age of the women was approximately 29 years for each survey. A majority of women (76.7%) in 1990–91 were uneducated. This proportion decreased in each survey, reducing to 50.7% in 2017–18. The women were mainly not employed, showing proportions of about three-quarters or more for unemployment in all surveys (Table 1).

**Table 1:** Socio-demographic indicators of the current use and non-use of contraceptive among women in reproductive age in Pakistan (all PDHS, n = 25,415, weighted n = 25,318)

	Current use of contraceptives				Current non–use of contraceptives			
	1990–1991 (n = 521)	2006–2007 (n = 1574)	2012–2013 (n = 2674)	2017–2018 (n = 2783)	1990–1991 (n = 3501)	2006–2007 (n = 4132)	2012–2013 (n = 4697)	2017–2018 (n = 5436)
	f (%)	f (%)	f (%)	f (%)	f (%)	f (%)	f (%)	f (%)
Age (in years)								
15–25	128 (24.6)	331 (21)	651 (23.6)	583 (20.9)	1300 (37.1)	1448 (35.0)	1585 (33.7)	1848 (34)
26–34	282 (54.1)	887 (56.4)	1543 (55.8)	1623 (58.3)	1611 (46.0)	1931 (46.7)	2309 (49.2)	2677 (49.2)
36–49	111 (21.3)	356 (22.6)	570 (20.6)	577 (20.7)	590 (16.9)	753 (18.2)	803 (17.1)	911 (16.8)
Type of residence								
Rural	113 (21.7)	779 (50.5)	1507 (54.5)	1490 (53.5)	1903 (54.4)	2921 (70.7)	1771 (33.7)	2216 (40.8)
Urban	408 (78.3)	795 (49.5)	1257 (45.5)	1293 (46.5)	1598 (45.6)	1211 (29.3)	2926 (62.3)	3220 (59.2)
Region								
Punjab	219 (42)	775 (49.2)	905 (32.7)	696 (25.0)	1133 (32.4)	1519 (36.8)	1103 (23.5)	1030 (18.9)
Sindh	169 (32.4)	371 (23.6)	504 (18.2)	457 (16.4)	896 (25.6)	1250 (30.3)	1087 (23.1)	984 (18.1)
Khyber Pakhtunkhwa	107 (20.5)	322 (20.5)	518 (18.7)	516 (18.5)	936 (26.7)	791 (19.1)	1014 (21.6)	866 (15.9)
Balochistan	26 (5)	106 (6.7)	270 (9.8)	182 (6.5)	538 (25.3)	572 (13.8)	879 (18.7)	820 (15.1)
Gilgit Baltistan	–	–	264 (9.6)	246 (8.8)	–	–	445 (9.5)	368 (6.8)
Islamabad (ICT)	–	–	303 (11.0)	256 (9.2)	–	–	169 (3.6)	285 (5.2)
AJK	–	–	–	272 (9.8)	–	–	–	590 (10.9)
Federally Administered Tribal Areas	–	–	–	158 (5.7)	–	–	–	493 (9.1)
Wealth index								
Poor	–	376 (23.9)	758 (27.4)	835 (30.0)	–	2141 (51.8)	2348 (50)	2817 (51.8)
Middle	–	329 (20.9)	515 (18.6)	619 (22.2)	–	788 (19.1)	914 (19.5)	990 (18.2)
Rich	–	869 (55.2)	1491 (53.9)	1329 (47.8)	–	1203 (29.1)	1435 (30.6)	1629 (30.0)
Mother’s education								
No education	236 (45.3)	826 (52.2)	1148 (41.5)	1033 (37.1)	2851 (81.4)	2972 (71.9)	2973 (63.3)	3101 (57.0)
Primary	72 (13.8)	270 (17.2)	455 (16.5)	420 (15.1)	299 (8.5)	518 (12.5)	610 (13)	673 (12.4)
Secondary	181 (34.7)	300 (19.1)	648 (23.4)	739 (26.6)	318 (9.1)	459 (11.1)	725 (15.4)	1000 (18.4)
Higher	32 (6.1)	178 (11.3)	513 (18.6)	591 (21.2)	33 (0.9)	183 (4.4)	389 (8.3)	662 (12.2)
Respondent’s occupation								
Not working	450 (86.4)	1166 (75.6)	2213 (80.1)	2372 (85.3)	2965 (84.8)	2915 (72.1)	3723 (79.3)	4753 (87.5)
Skilled	10 (1.9)	180 (11.7)	234 (8.5)	94 (3.4)	215 (6.1)	521 (12.9)	559 (11.9)	216 (4.0)
Unskilled	41 (7.9)	127 (8.2)	40 (1.4)	191 (6.9)	281 (8.0)	530 (13.1)	76 (1.6)	341 (6.3)
Professional	20 (3.8)	69 (4.5)	277 (10.0)	124 (4.5)	37 (1.1)	79 (2.0)	338 (7.2)	125 (2.3)
Ethnicity								
Punjabi	–	568 (37.2)	738 (26.7)	–	–	1023 (25.7)	818 (17.4)	–
Sindhi	–	140 (9.2)	181 (6.6)	–	–	679 (17.0)	561 (11.9)	–
Pashto	–	342 (22.4)	583 (21.1)	–	–	930 (23.3)	1116 (23.8)	–
Balochi	–	40 (6.2)	81 (2.9)	–	–	274 (6.9)	269 (5.7)	–
Others	–	437 (28.6)	1179 (42.7)	–	–	1080 (27.1)	1931 (41.1)	–

In each sub-sample data was weighted, leading to differences in absolute values

Overall, 585 cases had missing values

### Association between socio-demographic factors and current non-use of contraceptives

Four data series illustrate that in the years 1990–1991, 2006–2007, 2012–2013 and 2017–2018, the proportions of women who did not currently use any contraceptive method were 86.9, 72.2, 63.0 and 65.6%, respectively. The highest use of contraceptives was observed in the age group of 26–34 years. A majority of women with a poor wealth index did not use contraceptive methods; this proportion remained almost constant, ranging from 51.8% in 2006–2007 to 50.0% in 2012–2013 and 51.8% in 2017–2018. The use of contraceptives in rich families decreased over time from 55.2% in 2006–2007 to 47.8% in 2017–2018. The non-use of contraceptive methods among illiterate women declined from 81.4% (1990–1991) to 71.9% (2006–2007), 63.3% (2012–2013) and 57.0% (2017–2018) (Table 1).

### Demand-side factors and current non-use of contraceptives

Data regarding media exposure and non-use of contraceptive methods showed decreasing trends from 63.4% in 2012–2013 to 54.9% in 2017–2018. Women aged 15–49 who desired (more) children and did not use contraceptive methods accounted for 64.6% in 1990–1991, 57.4% in 2006–2007, 53.4% in 2012–2013 and 70.8% in 2017–2018. In 2012–2013, 41.0% of women who had faced intimate partner violence did not use any contraceptive methods, whereas this number declined to 29.4% in 2017–2018. About 59.9% of women who had not faced any kind of violence used contraceptive measures in 2012–2013, and this percentage increased to 71.5% in 2017–2018. About 41.6% who did not have decisional autonomy did not use any contraceptive methods in 2012–2013, and there was an increase in this proportion to 71.8% in 2017–2018. About 30.8% of the women who did not use contraceptives reported that it was a big problem to attend any medical facility in 2012–2013; this figure remained almost constant and was 30.5% in 2017–2018. About 65.3% in 2012–2013 and 70.0% in 2017–2018 who did not want to go alone to seek medical healthcare and reported this as a problem never used any contraceptive measures. Among those women who did not have sons, 17.8% (1990–1991), 20.1% (2006–2007), 21.1% (2012–2013) and 23.1% (2017–2018) did not use any contraceptive methods; this shows an increasing trend of not using contraceptives over the years (Table 2).

**Table 2:** Frequency and weighted percentages of demand- and supply-side factors of the women who currently use and do not use contraceptive methods in Pakistan

	Current use of contraceptives				Current non-use of contraceptives			
	1990–1991 (n = 528)	2006–2007 (n = 1574)	2012–2013 (n = 2674)	2017–2018 (n = 2783)	1990–1991 (n = 3501)	2006–2007 (n = 4132)	2012–2013 (n = 4697)	2017–2018 (n = 5438)
	f (%)	f (%)	f (%)	f (%)	f (%)	f (%)	f (%)	f (%)
*Demand-side factors*								
Media exposure								
No	–	–	511 (18.6)	728 (26.2)	–	–	1715 (36.6)	2449 (45.1)
Yes	–	–	2238 (81.4)	2053 (73.8)	–	–	2966 (63.4)	2982 (54.9)
Desire for more children								
No	392 (75.4)	1010 (64.2)	1732 (62.9)	1626 (58.6)	1218 (35.4)	1757 (42.6)	2150 (46.6)	1557 (29.2)
Yes	128 (24.6)	563 (35.8)	1022 (37.1)	1148 (41.4)	2225 (64.6)	2364 (57.4)	2460 (53.4)	3775 (70.8)
Intimate partner violence								
No	–	–	489 (59.9)	581 (71.5)	–	–	722 (59.0)	1011 (70.6)
Yes	–	–	327 (40.1)	232 (28.5)	–	–	501 (41.0)	420 (29.4)
Decisional autonomy								
No	–	–	487 (25.1)	1243 (66.5)	–	–	1270 (41.6)	3166 (71.8)
Yes	–	–	1450 (74.9)	820 (33.5)	–	–	1780 (58.4)	1631 (28.2)
Permission to go for medical or health facility								
Big problem	–	–	410 (14.9)	564 (20.3)	–	–	1442 (30.8)	1656 (30.5)
Not a big problem	–	–	2347 (85.1)	2217 (79.7)	–	–	3247 (69.2)	3776 (69.5)
Getting medical help for self: not wanting to go alone								
Big problem	–	–	1298 (47.1)	1617 (58.1)	–	–	3061 (65.3)	3800 (70.0)
Not a big problem	–		1459 (52.9)	1164 (41.9)	3061 (65.3)	3061 (65.3)	1628 (34.7)	1632 (30.0)
Having sons								
No	53 (10.2)	167 (10.6)	363 (13.1)	340 (12.2)	622 (17.8)	823 (20.1)	993 (21.1)	1257 (23.1)
1–3	366 (70.2)	1145 (72.7)	2078 (75.2)	2174 (78.1)	2359 (67.4)	2779 (67.3)	3219 (68.5)	3731 (68.6)
4–6	85 (18.2)	249 (15.8)	311 (11.3)	263 (9.5)	488 (13.9)	495 (12.0)	451 (9.6)	424 (7.8)
7–10	7 (1.3)	13 (0.8)	12 (0.4)	6 (0.2)	32 (0.9)	26 (0.6)	34 (0.7)	24 (0.4)
*Supply-side factors*								
Distance to health facility								
Not a big problem	–	–	2381 (50.8)	3008 (55.4)	–	–	856 (31.0)	3008 (55.4)
Big problem	–	–	2308 (49.2)	2423 (44.6)	–	–	1901(69.0)	2423 (44.6)
Transport availability								
Not a big problem	–	–	2621 (55.9)	–	–	–	934 (33.9)	–
Big problem	–	–	2067 (44.1)	–	–	–	1823 (66.1)	–
Visited by family planning worker in past 12 months								
No	–	–	1526 (53.0)	1112 (40.0)	–	–	860 (44.1)	2826 (52.0)
Yes	–	–	1353 (47.0)	1671 (60.0)	–	–	1089 (55.9)	2609 (48.0)
Unmet need								
No	–	1574 (100.0)	–	0 (0.0)	–	2915 (48.2)	–	2906 (55.7)
Yes	–	0 (0.0)	–	2777 (100.0)	–	2059 (51.8)	–	2310 (44.3)

### Supply-side factors and current non-use of contraceptives

Among supply-side factors, of those women who considered distance to a health facility a big problem, 69.0% in 2012–2013 and 44.6% in 2017–2018 never used any contraceptive measures. Of those who did not consider access to a health facility a big problem, 50.8% in 2012–2013 and 55.4% in 2017–2018 used contraceptive measures. Of those who considered transportation a big problem, 66.0% in 2012–2013 did not currently use contraceptive methods. In 2012–2013, 47.0% and in 2017–2018, 60.0% of women who were visited by family planning workers used contraceptive measures. Of those who were not visited by family planning workers, 44.1% in 2012–2013 and 52.0% in 2017–2018 did not use contraceptive measures. In 2006–2007, 48.2% of those who had unmet needs for contraception never used any contraceptive measures and this percentage increased to 55.7% in 2017–2018 (Table 2).

### Association between demand and supply-side factors and non-use of contraceptives

#### Demand-side factors

The odds of non-use of contraceptive methods were significantly lower among women who reported any source of media exposure in 2012–2013 (OR = 0.396, 95% CI 0.353–9.442) and in 2017–2018 (OR = 0.432, 95% CI 0.391–0.477) compared to those who did not have any media exposure. The odds of not using contraceptive methods were significantly higher for respondents who reported a desire for more children in all four waves of data collection. Respondents with decisional autonomy were more likely to use contraceptive methods. Similarly, the likelihood of using contraceptive methods was significantly higher among the respondents who had permission to attend medical or health facilities (Table 3).

**Table 3:** Association between non-user of contraceptives with demand- and supply-side factors

	OR (95% CI)				AOR (95% CI)			
	1990–1991	2006–2007	2012–2013	2017–2018	1990–1991	2006–2007	2012–2013	2017–2018
*Demand-side factors*								
Media exposure	–	–	0.396 (0.353–0.442)	0.432 (0.391–0.477)	–	–	0.664 (0.562–0.784)	0.654 (0.483–0.885)
Desire for more children	5.594 (4.528–6.911)	2.414 (2.140–2.722)	1.939 (1.761–2.136)	3.434 (3.120–3.780)	4.835 (3.803–6.147)	4.817 (3.972–5.841)	0.838 (0.627–1.119)	0.860 (0.632–1.161)
Intimate partner violence	–	–	1.038 (0.866–1.243)	1.040 (0.860–1.258)	–	–	0.801 (0.625–1.026)	0.875 (0.658–1.164)
Decisional autonomy	–	–	0.471 (0.415–0.534)	0.781 (0.702–0.869)	–	–	0.928 (0.799–1.077)	0.853 (0.647–1.125)
Permission to go for medical or health facility	–	–	0.393 (0.348–0.444)	0.580 (0.520–0.647)	–	–	0.729 (0.606–0.870)	1.238 (0.888–1.727)
Getting medical help for self: Not wanting to go alone	–	–	0.473 (0.430–0.521)	0.597 (0.543–0.656)	–	–	0.901 (0.780–1.040)	1.119 (0.816–1.534)
Having sons								
No	0.390 (0.164–0.925)	0.401 (0.202–0.797)	1.036 (0.531–2.022)	1.082 (0.439–2.668)	0.692 (0.262–1.830)	0.112 (0.022–0.583)	0.900 (0.413–1.961)	0.271 (0.150–0.490)
1–3	0.709 (0.311–1.619)	0.824 (0.422–1.609)	1.829 (0.945–3.540	2.331 (0.951–5.711)	1.280 (0.534–3.073)	0.211 (0.041–1.076)	1.574 (0.743–3.338)	1.516 (0.846–2.716)
4–6	0.890 (0.382–2.076)	1.006 (0.508–1.992)	1.954 (0.996–3.833)	2.481 (1.001–6.150)	0.923 (0.376–2.263)	0.323 (0.063–1.663)	1.795 (0.840–3.834)	1.779 (0.985–3.216)
*Supply-side factors*								
Distance to health facility	–	–	0.436 (0.395–0.482)	0.552 (0.503–0.606)	–	–	0.775 (0.656–0.916)	0.773 (0.554–1.078)
Transport availability	–	–	2.474 (2.244–2.728)	–	–	–	0.654 (0.555–0.777)	–
Visited by family planning worker in past 12 months	–	0.583 (0.514–0.660)	1.428(1.271–1.603)	0.614 (0.560–0.674)	–	0.668 (0.558–0.800)	0.732 (0.651–0.823)	0.631 (0.480–0.828)

Analysis is adjusted for age of mother, type of residence, region, mothers’ education, wealth, and occupation

Binary logistic results also indicate that the odds of not using contraceptive methods were significantly lower among respondents who reported fewer than 7 sons compared to the respondents who reported having 7 or more sons in 1990–1991. This association was insignificant in 2006–2007. However, in 2012–2013 and 2017–2018, the odds of not using contraceptive methods was higher among the respondents who reported 0–6 sons compared to participants with 7 or more (Table 3).

#### Supply-side factors

The results for supply-side factors also indicate that the odds of not using contraceptive methods were significantly lower among respondents who reported distance from a health facility in 2012–2013 (OR = 0.436, 95% CI 0.395–0.482) and 2017–2018 (OR = 0.552, 95% CI 0.503–0.606). The likelihood of not using contraceptive methods was significantly higher among respondents who reported the availability of transport facilities (OR = 2.474, 95% CI 2.244–2.728) in 2012–2013. The odds of not using contraceptive methods were significantly lower among respondents who reported a visit by a family planning worker during the previous 12 months in 2006–2007 (OR = 0.583, 95% CI 0.514–0.660) and 2017–2018 (OR = 0.614, 95% CI 0.560–0.674). However, the odds of not using contraceptive methods were significantly higher among respondents who reported a visit by a family planning worker during the previous 12 months in 2012–2013 (Table 3).

### Multivariable results

Table 3 also includes the results of a binary logistic regression analysis. These results indicate that the odds of not using contraceptive methods are higher among respondents who had exposure to any source of information compared to respondents who did not have any media exposure, both in 2012–2013 (AOR = 0.664, 95% CI 0.562–0.784) and 2017–2018 (AOR = 0.654, 95% CI 0.483–0.885). The odds of not using contraceptive methods was higher among respondents who reported a desire for more children in 1990–1991 (AOR = 4.835, 95% CI 3.803–6.147) and 2006–2007 (AOR = 4.817, 95% CI 3.972–5.841). However, the desire for more children lost significance in 2012–2013 and 2017–2018.

Multivariable results indicate that the odds of using contraceptive methods were lower among respondents who reported that they had permission to attend a medical or health facility in 2012–2013 (AOR = 0.729, 95% CI 0.606–0.870) compared to respondents who reported that they did not have permission. However, this variable lost its significance in 2017–2018. The odds of using no contraceptive methods were lower among respondents who reported a distance from a health facility (AOR = 0.775, 95% CI 0.656–0.916) compared to respondents who did not report a distance from a health facility in 2012–2013. However, it lost its significance in 2017–2018. The results also indicated that non-availability of transport (2012–2013) and limited visits by family planning workers over the previous 12 months (2006–2007, 2012–2013 and 2017–2018) were significant factors for not using contraceptive methods. On the other hand, a desire for more children, intimate partner violence, decisional autonomy, getting medical help and the number of sons lost significance in the multivariable analysis in 2012–2013. Similarly, a desire for more children, intimate partner violence, decisional autonomy, permission to attend a medical or health facility, getting medical help and the distance from medical care lost significance in 2017–2018 (Table 3).

## Discussion

The use of contraceptives is considered to be a mechanism for reducing poverty [32] and a tool to achieve human rights for women in terms of equitable health facilities, allowing decisional autonomy and control over their reproductive rights [33, 34]. However, in spite of efforts to increase contraceptive use, high fertility rates and population growth have continued to threaten the socio-economic development of many countries [35]. Various interventions have been introduced to address this issue over a significant period of time, i.e. awareness campaigns, celebrity appeals, appeals through religious scholars, access to free services and door to door services through LHWs. This study is based on a trend analysis of four PDHS data collections to determine the trends in demand- and supply-side factors associated with the non-use of contraceptives in Pakistan, along with socio-demographic variables.

The study found that socio-demographic factors play a significant role in the use of contraceptive measures. Those who live in rural areas are less likely to use contraceptive measures, with a trend that is increasing over time. These findings are similar to those of studies conducted by Adebowale et al. [36], who stated that geographical location limits access to family planning services. Rural residents have less access to health facilities. This trend is the same in this province-wide analysis as provinces where health facilities are better and accessibility issues are lower, greater prevalence of contraceptives have been observed. A greater use of contraceptives in Punjab province is observed due to better health facilities and more awareness campaigns compared to other provinces [24, 37–46].

Comparing results across the years 1990–1991, 2006–2007, 2012–2013 and 2017–2018, it is pertinent to note that there is a declining trend of not using contraceptives among illiterate women. The majority of women with a higher level of education are using contraceptive methods and this trend has increased over the years. The findings confirm those indicated by previous research that higher fertility is associated with a lower level of education [38, 39]. The wealth quintile is also significantly linked with the ever-use of contraceptive methods. Those in the poor quintile use contraceptive methods less than those in other quintiles. However, there is a rise in not-working women who have used contraceptives. Similar findings appear in a study conducted by Islam et al. [40], which indicates that the use of contraceptive measures among unemployed women in Bangladesh was lower than among employed ones.

A higher prevalence of contraceptive measures is found among women who have more knowledge, awareness, and media exposure [41, 42]. Our findings are consistent with these studies, as those women (aged 15–49) who have greater media exposure have more knowledge about contraceptive measures, and the use of contraceptives measures among them is also more likely [47]. Ethnicity is also significantly associated with the ever-use of contraceptive measures [43]. In our study, the use of contraceptives among different ethnic groups was higher among Punjabi women.

The findings of our study also suggest the intuitive results, that a greater desire for children is associated a lower likelihood of using contraceptive measures [24]. This is also associated with the women’s decisional autonomy; those women who had more decisional autonomy in their household and health-related decision-making power use contraceptives more than women who do not have autonomy in making decisions [44]. Therefore, gender disparity, lack of power in decision-making, discussions related to health issues, and limited permission to attend medical or health facilities are socio-cultural factors that also negatively impact on the use of contraceptives [45, 46]. Our findings show that women who face problems in attending health facilities are less likely to have ever used any method of family planning.

The supply side requires a considerable effort to provide adequate accessibility and resources of contraceptive measures [46]. Our findings show that there is a significant association between the distance to health facilities and the availability of transport with current use of contraceptive measures. Among those who have access to healthcare facilities, have transport facilities and are being visited by family planning workers, the use of contraceptive measures is more likely. It highlights the need for the provision of family planning resources and further structural factors, particularly in remote areas. More interventions are required to increase the knowledge and practice of use of contraceptives. The study provides a baseline for further research to find out the feedback of the LHWs, the way that interventions can change the behaviour and the attitude of people that may help in the attainment of reproductive health goals.

### Limitations

This study involves the data series provided by four PDHSs, in which a lack of uniformity was found because not all variables were included in all datasets, leading to missing variables in PDHS 1990–1991 and 2006–2007. A lack of uniformity was also observed in the variables related to ethnicity, wealth and women’s occupation in three sets of data. Therefore, the analysis remains limited to one or two datasets. Causal relationships cannot be determined due to the cross-sectional study design. A risk of bias is pertinent because information was gained through self-reported measures.

### Conclusion

This study concludes that although the use of contraceptive methods has increased over time, the rate of growth is inconsistent. Several socio-demographic characteristics and demand-side factors, such as media exposure, a desire for more children and the decisional autonomy of mothers, are associated with the use of contraceptives. Among these factors, differences are evident in rural, illiterate and non-working mothers. Access to family planning resources, the availability of transport, a supply of contraceptives in the locality and unmet needs are the associated supply-side factors found in this study. Overall, the results highlight a need to provide affordable family planning services to women near their homes, especially for disadvantaged subgroups of women where even LHWs have limited access. Targeted community mobilisation and increasing educational levels among mothers in rural communities can further increase the awareness about and use of contraceptives.

## Data Availability

Secondary data, available from the Demographic and Health Survey programme (https://dhsprogram.com/).

## Abbreviations

AOR: Adjusted odds ratio
CI: Confidence interval
LHW: Lady health worker
OR: Odds ratio
PDHS: Pakistan Demographic and Health Survey
SPSS: Statistical Package of Social Science
